# Bounded Rationality and Heuristics: Do We Only Need to Score in Order to Win Individual Awards in Basketball?

**DOI:** 10.3390/ijerph19042383

**Published:** 2022-02-18

**Authors:** Zsombor Zilinyi, Ágoston Nagy, Szilvia Borbély, Tamás Sterbenz

**Affiliations:** 1 School of Doctoral Studies, Hungarian University of Sports Science, 1123 Budapest, Hungary; 2 Sports Science Coordination Institute, University of Debrecen, 4032 Debrecen, Hungary; nagoston@sport.unideb.hu; 3 Institute of Physical Education and Sports Studies, University of Nyíregyháza, 4400 Nyíregyháza, Hungary; urbinneszilvi@gmail.com; 4 Sport Economics and Decision Making Research Centre, Hungarian University of Sports Science, 1123 Budapest, Hungary; sterbenz.tamas@tf.hu

**Keywords:** bounded rationality, heuristics, anchoring effect, points, basketball players

## Abstract

In the game of basketball game-related statistics are utilised to help decision makers to evaluate players’ achievements. Previous research showed that in the case of individual awards, points are preferred over other indicators of effectiveness. Based on recent studies and following Simon’s bounded rationality, in our interpretation, decision-makers decide on nominations at the post-grant level according to points scoring which is the easiest aspect to assess and the most familiar to them. In this context we also hypothesise that youth all-star players have better overall performance than their not selected teammates. To test our hypotheses we selected all of the youth awarded male players and their teammates from 2004 to 2019. In our sample, we examined *n* = 3198 player statistics. Two groups were created with nominated and not selected players. We used a two-sample *t*-test, and correlation matrix to examine the relationship between the variables (*p* < 0.001). We found that scoring is the most important selection criteria for decision-makers (r = 0.605; *p* = 0.000) and the selected players had significantly better individual statistics. An important finding of our study is that although efficiency (EFF) is used to measure the players’ contribution to the game, it is not the primary selection factor and should therefore be redefined.

## 1. Introduction

Talent identification and management focuses on measuring and comparing the characteristics that influence performance. In order to screen out less talented individuals, researchers often compare different age groups and education levels using cross-sectional studies [1]. This type of methodology is based on the assumption that important traits can be filtered out from individual performance over a period of time [2]. This element of thought conceives of talent as static and unchanging, and takes less account of other factors. Such factors may include, for example, the process of maturation, adolescence, and the relative age effect [3].

Analysing the literature, we can distinguish three levels of talent research. At the first level, we find research related to cognitive and psychological abilities. If we classify research on talent, the second type includes research on the physical profile: anthropometry, physiological and motor skills. The third type of talent research includes those factors that we have examined, which are based primarily on effectiveness and previous experience. These types of studies focus on the extent to which performance indicators, results and competitive rankings determine later predicted performance.

There are many factors that influence talent management that are not, or only marginally, addressed in the current literature. Relevant research focuses on the development of complex talent selection models and talent identification (TID) programmes [4].

The basketball literature also addresses direct, effectively measurable factors related to talent identification and selection; game-related statistics assist decision-makers in team and player evaluations. Most research related to team performance and predicting the outcome of a game or tournament tends to focus on the critical factors that differentiate winning and losing teams and determine the outcome of a game or tournament [5,6,7,8,9,10]. Some studies have identified field goal percentage, defensive rebounds [11,12,13], and assists [12] as game-related statistics that are highly correlated with success [11]. Assists, steals, and blocks are also important factors when considering key determinants of winning [14,15]. According to winning, there were studies which pointed out important discriminating factors, such as age [16], gender [17], and the locations of the games [18].

If there are game-related statistics which can underline the difference between wins and losses with scientific rigour, do we choose players who are the best in these statistical contexts? From another point of view, some North American research proves that in the case of individual awards, player salaries, all-star voting, and scoring as a statistical indicator is what decision-makers and professionals take into account, with emphasis on other indicators of efficiency. Berri, Brook, and Fenn [19] asked decision makers which game-related statistics they used to select potential players from universities by NBA teams. Based on university player statistics, the point/minute ratio has the greatest impact on selection. In contrast, the shooting percentage has a relatively low effect on the draft position, moreover rebounds and turnovers have virtually no effect on the position on the player’s exchange. However, the age of the player and the fact that the player’s team was among the top four in the college league were of paramount importance in the draft order.

Berri, Brook, and Schmidt [20] summarized coaches’ votes for the best NBA rookies from 1995 to 2007 and found that points scored were the most important statistical indicators for nominations, while experts also rated shooting percentage, rebounds, and turnovers as statistically significant. Berri, VanGilder and Fenn [21] came to a similar conclusion when analysing the votes and, as in the best rookie selection, in this statistical context the amount of points scored was the main determinant of the outcome of the MVP vote; however, among sports journalists, shooting efficiency was not a significant factor in the choice.

We were wondering whether this is a global or local mechanism in terms of selecting the best players. Can we detect a similar process in Europe for the European Youth Championships or are there other indicators dominating the selection? Do decision-makers settle down for one statistical dimension, or do they consider other elements as well? We believe that, in this case, bounded rationality and heuristics may come to the fore. Since people are not able to obtain or process all the information needed to make completely rational decisions, they instead seek to use the information available to them to achieve a satisfactory result [1]. Our cognitive limits set a barrier for us, and moreover personal relationships and social organizations also limit the decision-making, and so we have to strive to make satisfactory solutions. The “good enough” choices are, in most cases, satisfying; however this can lead sometimes to heuristic traps, such as systematic errors [22]. This raises the question in our research: can points as key statistical indicators cause systematic errors in our thinking, and are they dominating player selections in youth categories? Moreover, what are the most concrete statistics that influence the all-star squads in these tournaments?

In summary we hypothesised the following:

- As we assume cognitive bias [22,23] can play a role in decisions regarding sports, and we look for satisfactory solutions, points scored will be the best correlated variable with the entry into the all-star team between the statistical indicators of the players who have nominated. This is the most common statistical data, and thus decision-makers will choose this factor for evaluation.
- The game-related statistics of players who have joined the all-star team are better than those who have not been selected. These players have reached higher means than their teammates and they are significantly better in all statistical aspects.
- The amount of players selected from the championship finals into the all-star team is significantly higher than the players selected from less successful teams. These players have a better chance of selection as they play on the most important games where their possible exceptional performance can be weighted. Players from weaker teams cannot show up at the end, which can be a decisive factor, when judges select all-star players.
- Efficiency index (EFF) is a significant selection factor. This metric is intended to measure the relative performance of the player on the pitch.

While the “coach’s eye” works well most of the time in terms of selecting players, cognitive bias can lead to false interpretations of game-related statistics, too. There is a pressing desire to use the development of basketball statistics to inform our deeper decisions. Our goal is to observe certain statistical indicators in the youth categories that will both help us to understand the selection process and also to use these indicators going forward to help develop individuals and track key success factors to become respected players in their youth and, later, in their senior years. From another perspective, we would like to draw attention to decision-makers about possible opportunities for deeper evaluation of actual performance.

## 2. Materials and Methods

Unlike previous research on awards, our aim was to carry out an investigation about youth tournaments, as most of the recent studies mainly focus on senior sports. During the design of the study we wanted to clarify the terms related to the research. For a deeper understanding of our interpretation of youth tournaments we will shortly introduce the structure of the basketball federation’s competition.

In 2004, the International Basketball Federation’s (FIBA) tournament system underwent a major change, with the biennial European Youth Championships being replaced by a series of annual tournaments, renamed the U16, U18 and U20 European Championships. The cadet, junior and young adult age groups that had previously existed were replaced by the named age groups. As an important step in this restructuring, the national teams were ranked in divisions A and B, and from then on teams competed in a group stage and then in a cross-leg play-off and a knock-out play-off system for the best possible positions. Between these circumstances, players have more and more chances to compete. This large amount of data gave us the opportunity to work with a large representative sample.

In terms of the all-star players, we used the official designation. According to FIBA Regulations [24] youth awards are provided by the organisers of the youth tournaments and the Organising Committee decides the list of the awarded players. Five players are selected to the all-star team and one player is named as the MVP (Most Valuable Player). These prizes are given to the selected players in every youth tournament after the final game. There is no any specific rule, indication or limitation of the selection, and as such the judges have their own right to choose athletes for the ceremony.

To test our hypotheses and determine the key selection factors regarding all-star nominations, we intended to use the youth FIBA tournaments’ statistical database. These databases are publicly available and free to utilise. Similar to previous studies [25,26] we used FIBA archive pages and FIBA official tournament sites for data retrieval. Firstly we collected all of the former youth basketball players who have participated at youth European Championships and were selected to the all-star team or were named the ‘Most Valuable Player’ of the tournament. After we collected all of the publicly available individual statistics of the youth all-star national team players, we also gathered the statistics of the teammates of all-star national team players from the above mentioned websites. In our sample, we examined *n* = 3198 player statistics based on the statistical data of the 2004–2019 youth European Basketball Championships. The basketball players represented 36 countries. The sample was divided into U16 (28.1%), U18 (34.9%) and U20 (37%), in categories “A” (57.3%) and “B” (42.7%). These categories are called divisions, but in the current paper we did not aim to investigate the differences between these divisions. In the case of duplication of awards (for example a player got rewarded multiple times), we took the statistics of the older category into account. The data were cleaned and clustered with Microsoft Excel 2010, which was then imported to IBM SPSS Statistics. We have separated our sample into all-star selected (*n* = 320) and not selected (*n* = 2878) players.

As we indicated in the introduction section, we hypothesised that the all-star team would, in most cases, come from the national team winners of the European Championships, and we used two-sample *t*-tests to examine this. We analysed the whole sample with descriptive statistical indicators to find out whether there was an unusually high level of representation from one or more countries.

We investigated the sub groups’ (all-star, not selected) shooting statistics to search for significant differences. We determined the significance level at *p* < 0.005. In further analysis of all this, we also wanted to know which indicators would be significantly higher for the selected players and which ones would correlate better with making the all-star team selection. In doing so, we assumed that statistical data can be used to identify those variables that significantly influence selection. We have carried out a correlation test, the results of which therefore showed us the relationship between two variables.

## 3. Results

First of all, we examined the shooting statistics of the created sub-groups. As Table 1 Shows, shooting attempts are much higher in the case of all-star selected players than among those who were not selected. There is a strong significant difference for each variable (*p* < 0.001). In terms of the shooting statistics, the smallest difference between the means is the shooting percentages of field goals. On the other hand we can observe that the most significant difference between the all-star and non all-star players is the amount of field goal shots. In the case of the selected players, there were large variations in the standard deviation of shooting attempts, while not selected players’ values were lower.

**Figure 1:**
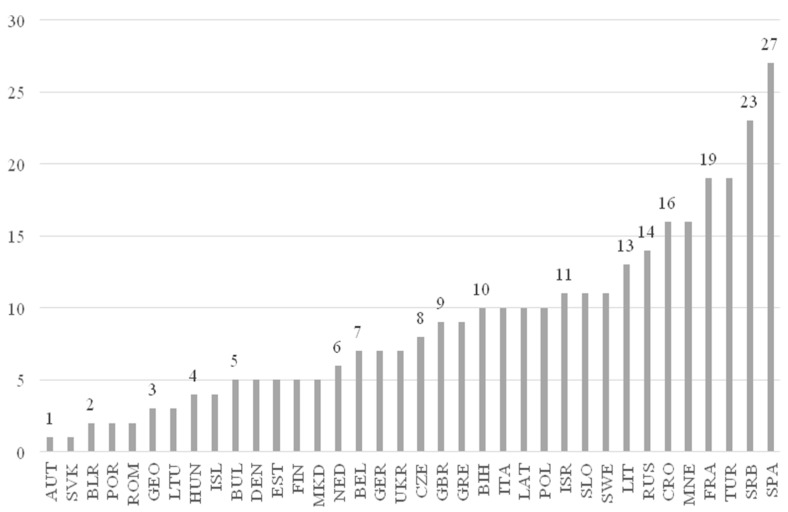
Number of players selected from nations (*n* = 320) (figure by the authors).

We also investigated whether we would find differences between the nationalities of the players selected for the all-star team, and whether there are any nationalities with a significantly higher proportion of players selected (Figure 1).

It was assumed that the all-star team would, in most cases, be made up of members of the team that won the championship, i.e., that the number of matches would show a higher correlation with the all-star team selection.

To examine this, we used a two-sample *t*-test. The results showed that we were able to detect a significant difference for both variables. This means that those who made the top five played an average of 7.92 games. Those who were not selected played on average 7.20 matches (t = −10.759, *p* = 0.000). A significant difference was also found when examining playing time (t = −37.050, *p* = 0.000). The average minutes played by the players who were selected among the top performers (mean = 226.76) is significantly higher than those who were not selected (mean = 124.15).

We also examined the differences in terms of rankings. We established four groups (1st–2nd place: first group; 3rd–4th place: second group; 5th–8th place: third group; 9th–16th place: fourth group). It can be seen that 55.9% of the players awarded played in the final of the tournament and a further 32.2% of the players were also in contention for the podium (t = 2.153 *p* = 0.031). These results imply that most of the winning members of the winning teams are selected for the all-star team.

The other elements of the statistical variables were also examined using the same method. It was found that for all variables the means were significantly higher for the selected players. This result suggests that the shooting percentages, offensive and defensive performance of the all-star team members are better in all aspects than those who were not selected.

In further analysis of all this, we also wanted to know which indicators would be significantly higher for the selected players and which ones would correlate better with making the team. In doing so, we hypothesise that statistical data can be used to identify those variables that significantly influence selection. For this purpose, a correlation test was carried out, the results of which show the relationship between the two variables. In all cases, inclusion in the all-star team is one of the variables in the study (Table 2.)

In the case of the item numbers, a lower value is observed for the efficiency (EFF) and the plus minus (+/−), since these statistical factors are not included on the previous statistical sheets directly. It can be observed that more significant correlations are the sum of points scored (PTS) (r = 0.605), the efficiency indicator (EFF) (r = 0.579) and the defensive rebounds (DREB) (r = 0.492) and attempts of two-point shots (2PA) (r = 0.480). There is also a medium relationship between the statistical indicators of field activities and playing time (MIN) (r = 0.422). An interesting result is that, although the difference between the two groups appears significant for all variables, the strength of the relationships is weak for shooting percentages, number of games (GP) and personal fouls (PF) (r < 0.250).

## 4. Discussion

Recent research has showed that scoring is a crucial factor for winning an individual award [18]. Our paper also confirmed the results of previous research. As far as the statistics of points are at the beginning or at the end of an official statistical sheet—and, it should be pointed out, this is the most frequently cited statistical data—it is presumably the most influential on selection decision-making processes. In the sense of bounded rationality, we are looking for satisfactory solutions, so scoring as a statistical factor stops further thinking in most cases.

From a player’s perspective, this conclusion can encourage opportunistic play, despite the fact that this behaviour is in contrary to the interests of the team. It is inevitable that young players have to be entrepreneurial, because this is the way for them to get attention from the decision-makers, and the opportunity to be selected after the finals.

Another remarkable finding of our study is that shooting efficiency is not as important as scoring in connection with youth all-star selection, although other studies have shown its importance considering team-winning [27,28]. Players’ shooting form may influence team performance, but this is not a significant decisive factor considering all-star nomination based on our research. We did not examine the semi-finals and finals separately, only the performance throughout the entire tournament, however an interesting line of research could be to analyse the performance of all star players in the most important matches.

The efficiency metric (EFF) is the second most significant selection factor. Despite the fact that this game-related statistic directly measures player efficiency, the decision-makers choose scoring over EFF. This can result in tensions between players and teams, and questions the relevance of the efficiency indicator. Previous studies have highlighted the problematic nature of the efficiency indicator. The following problems with EFF can be observed [29]:

- The indicators used in EFF are for absolute performance and there is no analysis of concrete efficiency.
- The absolute value of the indicators largely depends on the tempo of the game and the number of attacks.
- Measured game elements are estimated without weighting or processing empirical data into statistical indicators, so the evaluation function is biased.
- A decisive factor for performance cooperation, which is crucial for performance, is not evaluated by the EFF statistic, thus allowing opportunistic behaviour (selfish play).

This anomaly can be resolved by implementing a new statistic factor that measures efficient performance better. There have been research attempts to develop an efficiency indicator [29,30,31,32], but these indicators are not used on official FIBA tournaments. In order to make professional decisions that are acceptable to all, we also believe that the official statistical sheets need to be renewed.

Statistically defensive rebounds are also key success factors both in terms of team and individual success, while in our study the number of minutes played were not as important as we supposed them to be in connection with entering the all-star team.

In terms of the limitations of our study, all-star player selection can be biased on other not negligible factors, such as:

- nationality
- team rankings
- home-team advantage
- multiple selections from the past
- strong lobby activity
- tournament-ending injuries, resting during games, etc.

Although these players had a better overall statistical performance than their not selected teammates, in some ambiguous cases the nomination process can be based on non-statistic circumstances. These presence or absence of these factors can be an aim of further investigations regarding talent selection.

Prized players usually have a huge, comprehensive skill set, and in most cases decision-makers recognise talent, however it can be a danger to reduce the attention (even unintentionally) to one statistical dimension, such as points scored. If we make this mistake there is a chance of disregarding players with high shooting efficiency or rebounding and passing abilities. If awards affect young players’ professional future, decisions for nominating players should be based on deeper analysis.

## 5. Conclusions

All of our hypotheses were confirmed. Players with better statistical individual performance had a significantly higher chance of all-star nomination. Points, efficiency and defensive rebounds were the key statistical indicators on selection for the all-star teams. Despite the fact that EFF’s purpose is to measure a player’s utility, it is not the top selection criterion, however it is still a relevant game-related statistic. Similar to previous studies, points scored was the most dominant distinguishing factor in terms of all-star selection. This can be a heuristic trap, which could be avoided by rule changes and setting standards in connection with all-star nomination. From the players’ perspectives, current selection methods support opportunism; the results suggest that players ought to just score in order to win awards. Minutes played did not affect the selection process significantly according to our results, despite the fact that players with few minutes on court do not have real chance to get selected for prizes. As our results highlighted, players with successful teams have greater opportunities, as the winners and podium members are nominated sooner than players with poor team results.

We found a relatively low importance of shooting percentages in connection with selection, although in senior years a positive trend in the improvement of throwing percentages can be detected [33]. Earlier research [34] highlighted that 58% of the former all-star selected players were able to attend their senior team’s squad at least once. This data can encourage decision-makers to use the most appropriate statistical data for selection to the all-star team, as it can boost youngster’s careers. From this aspect, further analysis is needed in order to help informed decisions regarding player selection methods. To identify further pitfalls, we consider it worthwhile to analyse the selection of all star players by positions in the future.

## Figures and Tables

**Table 1:** Shooting attempts and accuracy of all-star and not selected youth basketball players (figure by the authors).

	All Star (1)—Not Selected (0)	*n*	Mean	Std. Deviation	Std. Error Mean	t	sig
FGA	0	2878	29.70	24.80	0.46	22.941	<0.001
FGA	1	320	77.28	36.17	2.02		
FG%	0	2878	40.34	14.59	0.27	12.090	<0.001
FG%	1	320	46.81	8.24	0.46		
2PA	0	2878	20.31	17.85	0.33	22.550	<0.001
2PA	1	320	54.79	26.69	1.49		
2P%	0	2878	45.43	18.16	0.33	11.237	<0.001
2P%	1	320	52.15	8.82	0.49		
3PA	0	2878	9.49	11.90	0.22	12.571	<0.001
3PA	1	320	23.02	18.84	1.05		
3P%	0	2878	21.97	19.72	0.36	8.341	<0.001
3P%	1	320	29.29	14.25	0.79		
FTA	0	2878	10.35	10.57	0.19	22.787	<0.001
FTA	1	320	30.65	15.53	0.86		
FT%	0	2877	56.14	30.78	0.57	14.028	<0.001
FT%	1	320	69.13	13.00	0.72		

Abbreviations: FGA-Field Goal Attempts, FG%-Field Goal Percentages, 2PA-Two-Point Attempts, 2P%-Two-Point Percentages, 3PA-Three-Point Attempts, 3P%-Three-Point Percentages, FTA-Free Throw Attempts, Free-Throw Percentages.

**Table 2:** Correlation of statistical indicators with selection to the all-star team (figure by the authors).

Statistical Indicator	r =	*p* =	*n* =
PTS	0.605	0.000	3198
EFF	0.579	0.000	1103
DREB	0.492	0.000	3198
2PA	0.480	0.000	3198
FGA	0.479	0.000	3198
FTA	0.479	0.000	3198
REB	0.465	0.000	3198
MIN	0.422	0.000	3198
TO	0.417	0.000	3198
STL	0.360	0.000	3198
AST	0.347	0.000	3198
BLK	0.327	0.000	3198
OREB	0.324	0.000	3198
3PA	0.303	0.000	3198
PF	0.193	0.000	3198
+/-	0.278	0.000	1103
FG%	0.137	0.000	3198
FT%	0.131	0.000	3187
GP	0.126	0.000	3197
2P%	0.115	0.000	3198
3P%	0.113	0.000	3197

Abbreviations: PTS-Points Scored, EFF-Efficiency, DREB-Defensive Rebounds, 2PA-Two-Point Attempts, FGA-Field Goal Attempts, FTA-Free Throw Attempts, REB-Rebounds, MIN-Minutes Played, TO-Turnovers, STL-Steals, AST-Assists, BLK-Blocks, OREB-Offensive Rebounds, 3PA-Three-Point Attempts, PF-Personal Fouls, +/- Plus-Minus Rating, FG%-Field Goal Percentages, FT%-Free Throw Percentages, GP-Games Played, 2P%-Two-Point Percentages, 3P%-Three-Point Percentages.

## Data Availability

We used the FIBA archive pages and FIBA official tournament sites for data collection and retrieval. FIBA Europe online database: http://www.fibaeurope.com/pageID_RX-q8vRSHxY9gJr0XLBQf3.compID_YUjW-7-FJ,kK9s431Lyr41.season_2016.html (2020, accessed from 29 November 2020 to January 31 2021) FIBA archive database: https://archive.fiba.com/pages/eng/fa/p/fromseason/1930/toseason/2019/q//cid//_//events.html (2020, accessed from 29 November 2020 to January 31 2021)
